# Use-value and importance of socio-cultural knowledge on *Carapa procera* trees in the Sudanian zone in Mali

**DOI:** 10.1186/1746-4269-11-14

**Published:** 2015-03-02

**Authors:** Urbain Dembélé, Anne Mette Lykke, Yénizié Koné, Bino Témé, Amadou Malé Kouyaté

**Affiliations:** Institut d’Economie Rurale (IER), Rue Mohamed V, BP: 258, Bamako, Mali; Institut Supérieur de Formation et de Recherche Appliquée (ISFRA), N’Golonina, Rue 268, Porte 238, BPE: 475, Bamako, Mali; Department of Bioscience, Aarhus University, Vejlsoevej 25, DK-8600 Silkeborg, Denmark

**Keywords:** *Carapa procera*, Native oil trees, Ethnobotanical knowledge, Use value, West Africa

## Abstract

**Background:**

*Carapa procera* is a native oil tree species with multipurpose values traditionally exploited by the local population in Southern Mali. This study focused on the assessment of local knowledge about the use of *Carapa procera*.

**Methods:**

Semi-structured ethnobotanical questionnaires were conducted among the ethnic groups Senufo, Fulani and Bambara in two localities in the Sudanian zone in Mali. Use values among these ethnic groups and gender were evaluated.

**Results:**

This study showed that *Carapa procera* is a species with multiple uses and high use values. According to the consensus value for plant parts (CPP), the nuts constituted 57% of exploited plant parts followed by bark and leaves (12%), wood and roots (7%), mistletoes (4%) and gum (1%). The use diversity (UD) values of *Carapa procera* showed a high proportion of cosmetic (UD = 0.49) and therapeutic (UD = 0.36) uses. The UD for therapeutic uses was higher for ethnic groups in Ziékorodougou than in Niankorobougou. In contrast, the UD for cosmetic uses was higher for ethnic groups in Niankorobougou than in Ziékorodougou. Comparative analysis between ethnic groups revealed that the highest UD for cosmetic uses (0.63) was observed in the Bambara ethnic group, whereas the highest UD for therapeutic uses (0.39) was obtained in the Senufo ethnic group. The UD showed that cosmetic uses were higher for women than for men in both locations. Men in Ziékorodougou had the highest level of knowledge regarding plant parts used, forms of use and the specific reasons for using *Carapa procera*.

**Conclusion:**

This study highlighted the sociocultural importance of *Carapa procera*. In the light of its multipurpose uses, the promotion and enhancement of *Carapa procera* can provide significant socio-economic benefits to local people. In this perspective, it is necessary to implement conservation strategies and sustainable management through domestication of the species.

## Background

In the Sahelian countries, the majority of people live in rural areas and depend mainly on natural resources for subsistence and income generation [1–3]. Wild plants play important social, cultural, aesthetic and ethical roles for rural communities, as local people depend on them for food [4, 5], traditional medicine [6, 7], construction, handicrafts [8], cosmetics, forage and revenues [9, 10]. In recent years, local knowledge has been increasingly studied using quantitative ethnobotanical studies to identify plants with high nutritional, medical and/or commercial potentials likely to contribute to improving the livelihood of local populations. These studies have assessed the relationship between biological and cultural diversity, knowledge on vegetation change and the relative importance of natural resources for the local population [7, 11–13]. Thus, a number of quantitative methods have been developed to study the cultural importance of plant species and differences between different communities and social groups [14–18]. Quantifying plant use and local knowledge makes it possible to make useful comparisons between different groups of informants [10, 19–23].

In Mali, a number of studies have been conducted to show the socio-cultural, economic and ecological importance of plant species. These studies showed that origin [24], location [4, 25], commercialization [9, 26, 27] and gender [28, 29] interact to influence how people use the species. Following these previous studies, our work combines both qualitative and quantitative analytical and ethnobotanical tools to show ethnic and gender differences in use patterns of *Carapa procera*.

Thus, our study aims to assess the importance of uses of *Carapa procera* and how the use-value varies between different ethnic groups, gender and location and, thereby, identify potentials for new income generation activities and improved sustainable management and conservation of the species. Specifically, it comes to identify the exploited parts, assess the use categories, characterize the forms of uses and analyze the reasons of use *Carapa procera*.

## Methods

### Study area

This study was conducted during the years 2011–2012 in two villages, Ziékorodougou and Niankorobougou, in the Sikasso region of Mali for general surveys and in four other villages, Sougoumba, Soungoulasso, Founa and Bounou, for interviews with key informants.

**Figure 1:**
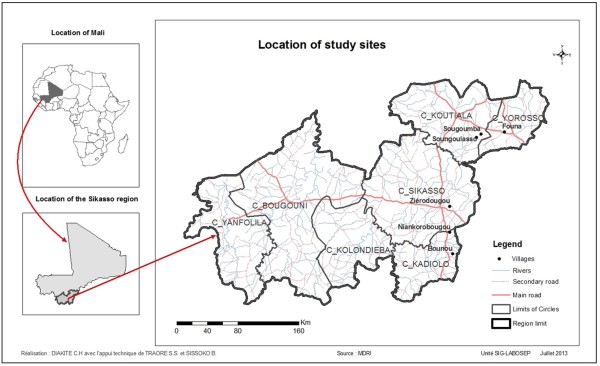
**Location of study area in Sikasso region, Mali.**

The Sikasso region is located in southeast Mali, 12° 30′ N and 8° 45′ W bordering Ivory Coast and Burkina Faso (Figure 1). The climate of the region is Sudanian to northern Guinean with 750 to 1400 mm [30]. The average annual temperature is 27°C. The vegetation of the region is mainly composed of woodland, wooded grassland, grassland, shrubland and gallery forest. The population of the region is mainly composed of following ethnic groups: Senufo, Minianka, Fulani, Samogo, Bobofing and Bambara.

### Study species

*Carapa procera* belongs to the Meliaceae family. In West Africa, three species are found: *Carapa microcarpa*, *Carapa procera* and *Carapa velutina*[31]. This study focused on *Carapa procera* DC., a woody species of Sudano-Guinean affinity growing along gallery forests and rocky faults [32]. *Carapa procera* is a large trees up to 30 m tall, occurring in a wide range of habitats from gallery forest in savanna areas to humid forest in western and central Africa [33]. Seed regeneration is the dominant form of species renewal [32]. *Carapa procera* is an oleaginous plant species traditionally exploited by rural communities in southern Mali. In the local language Bamanakan it is called “*kobi*”. *Carapa procera* plays an important role in the socio-economic and cultural life of local populations. The products are mainly used for oil and soap, because of their therapeutic and cosmetic properties. The oil extracted from nuts of *Carapa procera* has various medicinal applications, as insecticide and repellent and for veterinary care of animals. In addition to traditional uses, the oil is used for the phytosanitary treatment of organic crops such as cotton [32]. The leaves, bark and roots are also used in the treatment of some diseases as skin and gastrointestinal diseases [34]. Exploited products, particularly oil, are sold in local markets and bring substantial income to producers, especially women.

### Ethnobotanical surveys

Data collection was conducted using adapted semi-structured ethnobotanical questionnaires described by Lykke et al. [12]. Formal surveys were conducted in the villages of Ziékorodougou and Niankorobougou. The choice of these localities was based on the presence of a natural stands and local use of *Carapa procera* by local communities.

In each village, we held a first meeting with the village chief and his advisors to inform them of the purpose of our work. Two ethnic groups were chosen: Senufo and Fulani in Ziékorodougou, Bambara (Dioula) and Fulani in Niankorobougou. The survey covered 12 informants within each ethnic group of which six were men and six women (Table 1). The choice of informants was made in collaboration with village leaders. Informants were chosen randomly among those who have knowledge about *Carapa procera*. The interviews were conducted individually in the local language “Bamanakan”. The information collected during these investigations focused on general knowledge of *Carapa procera*, characteristics of the parts exploited, collection period, use categories and reasons for use. Here, a use category is defined by all uses of the same nature according to the method of Van Andel [1].

**Table 1:** **Number of informants by ethnic group**

Locality	Ethnic group	Number of informants	Age class
Ziékorodougou	Senufo	12	33–73
	Fulani	12	26–71
Niankorobougou	Bambara	12	20–65
	Fulani	12	28–62
**Total**		**48**	**20–73**

Supplementary interviews were conducted with other contact informants during prospective missions for more detailed information. Interviews were followed up by participant observations of processing and use of *Carapa procera* products according to Martin [17].

All participants were informed of the objectives of the study and that the results would be used for a scientific publication.

### Data analysis

The following ethnobotanical quantitative and qualitative methods were used for data analysis.

The frequency of exploited plant parts (F) from *Carapa procera* was evaluated through the response rate by type of plant part according to the formula:


S: the number of informants who responded positively to use a plant part; N: the total number of informants. F = 0 indicates that the plant part is not used; F is 100 when the part is used by all informants.

The formulas described by Monteiro et al. [35] were used to know the degree of agreement among informants on the plant parts exploited and the forms of use.

Consensus value for plant parts exploited (CPP):


P_x_: the number of citations of a plant part exploited; P_t_: the total number of citations of all plant parts.

Use values of *Carapa procera* were evaluated through the parameters that indicate how the exploited plant parts are used and how the knowledge of these uses is allocated among informants. The following formulas described by Byg & Baslev [36] were used.

Importance of use categories was evaluated through the use diversity value (UD) according to the formula:


U_CX_: the number of indications recorded by category of use; U_Ct_: the total number of indications for all categories of uses.

The use equitability value (UE) was calculated to know the degree of homogeneity of knowledge about use categories;


UD: use diversity value; UD_max_: the maximum value of the diversity index.

The degree of agreement among informants concerning the form of use was measured trough the formulas in [35]. Consensus value for the forms of use (CMU):


M_x_: number of citations for a form of use; M_t_: total number of citations for all forms of uses.

The fidelity level (FL) was calculated for the specific purposes of use of exploited plant parts following the formula of Friedman et al. [37]:


Where n is the number of informants for a specific use, and N is the total number of informants.

## Results

### Local names

In the study area, *Carapa procera* is identified through various local names (Table 2). The prefix of local names, “*ko*” in Bamanakan, “*dugu*” in Senufo and Minianka language means backwater and refers to the traditional habitat of the species. This shows that *Carapa procera* is a species traditionally listed along watercourses such as backwater and rivers.

**Table 2:** **Local names of** ***Carapa procera***

Ethnic group	Local name
Bambara	*kobi*
Fulani	*kobi*
Senufo	*gué, bii, dugugué*
Samogo	*fiè, firo*
Minianka	*douwè, duguwèrè, duguworo*

### Plant parts exploited

**Figure 2:**
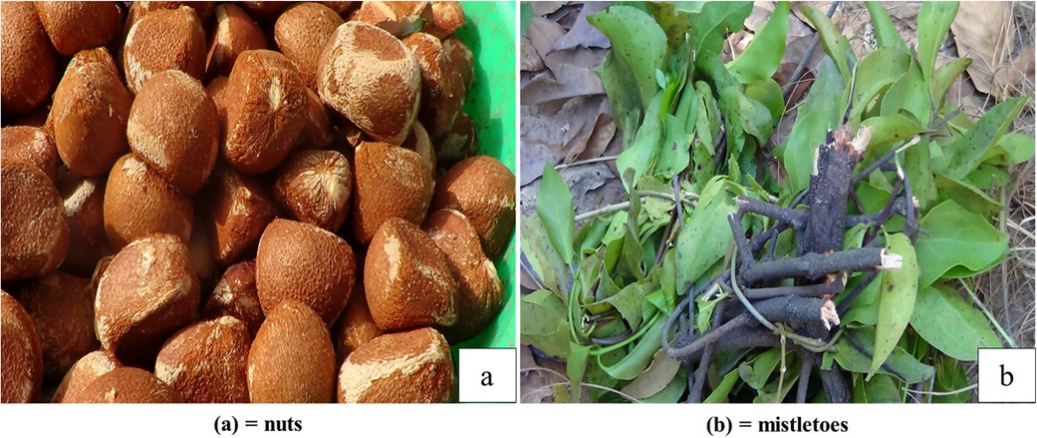
**Nuts and mistletoes exploited from** ***Carapa procera*** **.**

**Figure 3:**
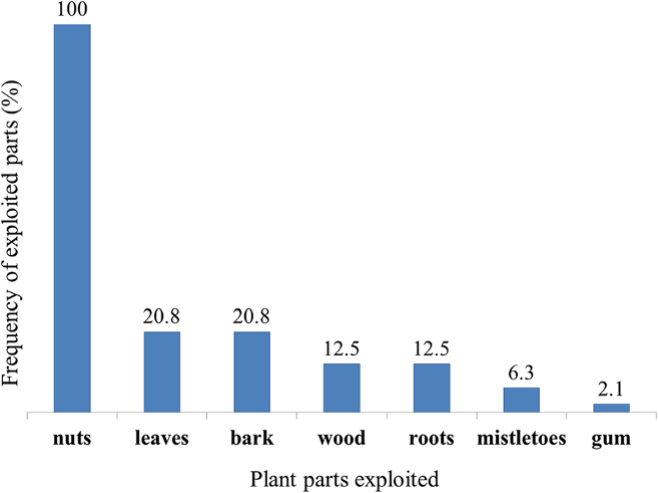
**Frequency (F) of exploited plant parts from** ***Carapa procera*** **.**

**Figure 4:**
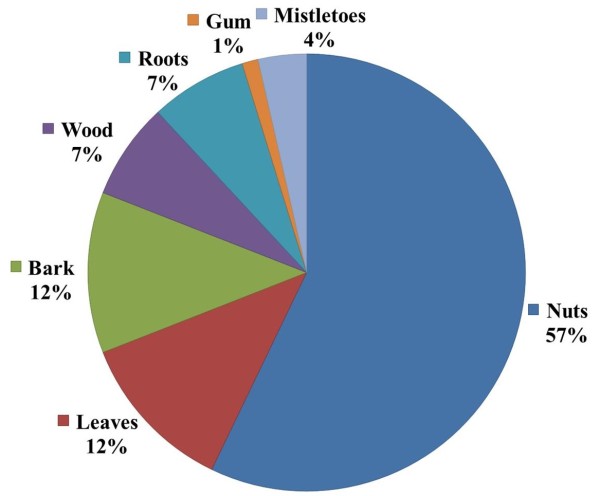
**Percentage of exploited plant parts from** ***Carapa procera*** **.**

**Figure 5:**
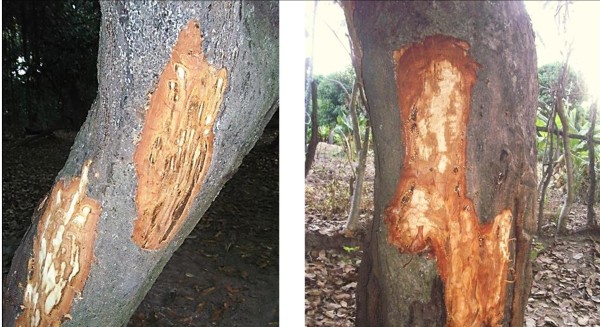
**Effects of bark exploitation of** ***Carapa procera*** **.**

Several plant parts of *Carapa procera* were exploited by local communities: roots, bark, wood, gum, leaves, nuts (Figure 2a) and mistletoes (Figure 2b). Mistletoes are parasitic plants from the Loranthaceae family growing on branches of some trees and collected for magical and spiritual purposes. The F indicates that nuts were mentioned by all informants (100%) followed by leaves and bark with 21% each (Figure 3). CPP showed that nuts constituted 57% of the citations of exploited plant parts, followed by bark and leaves (12%), wood and roots (7%), mistletoes (4%) and gum (1%) (Figure 4). Field observations have revealed the impact of the bark exploitation that may have adverse effects on the survival of the species (Figure 5). Nuts are usually collected at the beginning of the rainy season during the months from May to June. The other plant parts are exploited in any period.

Tables 3 and 4 show the consensus value for plant parts exploited (CPP) according to ethnic group and gender of informants. Nuts have obtained the highest consensus values irrespective of the ethnic group and the sex. Women showed a higher consensus value for nuts (0.71) than men (0.48). The consensus value for bark harvesting was higher for men (0.16) than for women (0.06) in Ziékorodougou. In Niankorobougou, the CPP for bark harvesting was the same (0.12) for both sexes.

**Table 3:** **Consensus value (CPP) for exploited plant parts from** ***Carapa procera*** **by ethnic group**

Part exploited	Ziékorodougou		Niankorobougou	
	Senufo	Fulani	Bambara	Fulani
Nuts	0.55	0.60	0.92	0.41
Leaves	0.09	0.10	0.08	0.17
Bark	0.09	0.15	-	0.17
Wood	0.09	0.10	-	0.07
Roots	0.09	-	-	0.14
Gum	0.05	-	-	-
Mistletoes	0.05	0.05	-	0.03

**Table 4:** **Consensus value for exploited plant parts from** ***Carapa procera*** **by gender**

Part exploited	Ziékorodougou		Niankorobougou	
	Men	Women	Men	Women
Nuts	0.48	0.71	0.48	0.71
Leaves	0.08	0.12	0.20	0.06
Bark	0.16	0.06	0.12	0.12
Wood	0.08	0.12	0.08	-
Roots	0.08	-	0.12	0.06
Gum	0.04	-	-	-
Mistletoes	0.08	-	-	0.06

### The use categories

**Figure 6:**
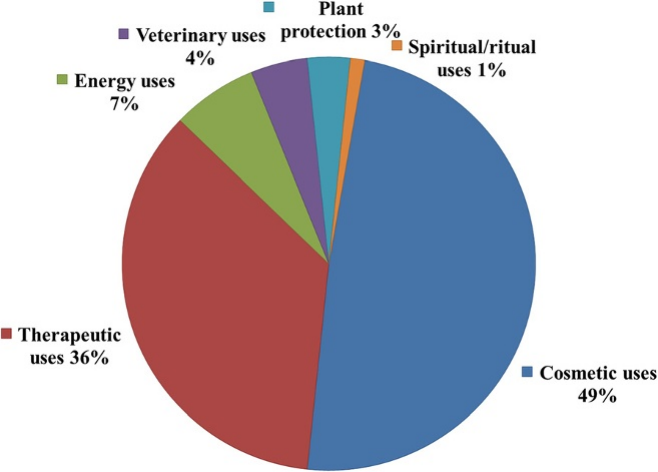
**Percentage of use categories from** ***Carapa procera*** **.**

The plant parts exploited are used for different purposes, which can be grouped according to use categories: therapeutic, cosmetic, veterinary, energy, plant treatment and ritual/spiritual. The cosmetic and therapeutic uses have got the highest percentage of use categories (49% and 36%, respectively) (Figure 6). Tables 5 and 6 show the UD and UE of use categories according to the ethnic groups and gender. Comparative analysis between ethnic groups revealed that the highest UD for cosmetic uses (0.63) was observed in the Bambara ethnic group, whereas the highest UD for therapeutic uses (0.39) was obtained in the Senufo ethnic group. The UD for therapeutic uses was higher for ethnic groups in Ziékorodougou than in Niankorobougou. In contrast, the UD for cosmetic uses was higher for ethnic groups in Niankorobougou than in Ziékorodougou. Veterinary uses were recorded particularly in Fulani ethnic group in both locations. Ethnic groups in Ziékorodougou reported uses of *Carapa procera* for plant protection. The Senufo ethnic group indicated spiritual and ritual uses of *Carapa procera*. Related to gender, the use diversity values showed that cosmetic uses were higher for women than for men. The therapeutic uses showed similar values for men in both locations (UD = 0.36). However, women in Ziékorodougou showed the highest value of therapeutic uses (UD = 0.40) in contrast to women in Niankorobougou, who presented the lowest value of therapeutic uses (UD = 0.30).

**Table 5:** **Use diversity values (UD) and use equitability values (UE) of use categories from** ***Carapa procera*** **according to village and ethnic group**

Category of use	Ziékorodougou				Niankorobougou			
	Senufo		Fulani		Bambara		Fulani	
	UD	UE	UD	UE	UD	UE	UD	UE
Therapeutic uses	0.39	0.88	0.36	0.82	0.32	0.51	0.35	0.73
Spiritual/ritual uses	0.04	0.09	-	-	-	-	-	-
Cosmetic uses	0.44	1	0.44	1	0.63	1	0.48	1
Veterinary uses	-	-	0.04	0.09	0.05	0.08	0.08	0.18
Plant protection	0.04	0.09	0.08	0.18	-	-	-	-
Energy uses	0.08	0.19	0.08	0.18	-	-	0.08	0.18

**Table 6:** **Use diversity values (UD) and use equitability values (UE) of use categories from** ***Carapa procera*** **according to village and gender**

Category of use	Ziékorodougou				Niankorobougou			
	Men		Women		Men		Women	
	UD	UE	UD	UE	UD	UE	UD	UE
Therapeutic uses	0.36	0.91	0.40	0.80	0.36	0.73	0.30	0.50
Spiritual/ritual uses	0.03	0.09	-	-	-	-	-	-
Cosmetic uses	0.39	1	0.50	1	0.50	1	0.60	1
Veterinary uses	0.03	0.09	-	-	0.05	0.09	0.10	0.17
Plant protection	0.11	0.27	-	-	-	-	-	-
Energy uses	0.07	0.18	0.10	0.20	0.09	0.18	-	-

### The forms of uses

**Figure 7:**
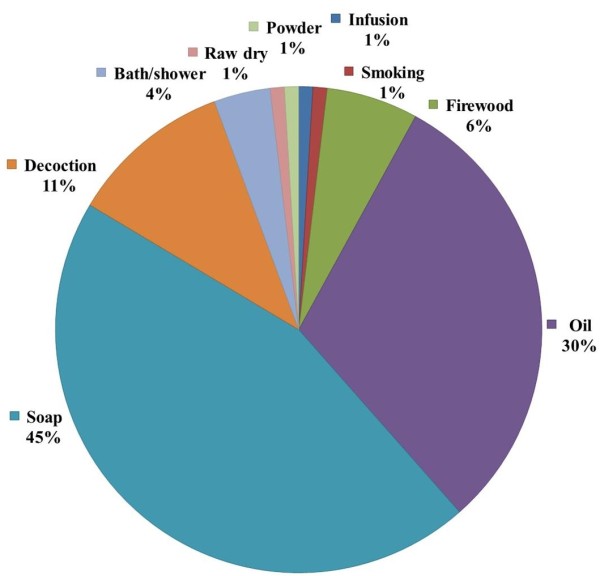
**Percentage of use forms of plant parts exploited from** ***Carapa procera*** **.**

Forms of use are varied. Leaves, roots or bark can be used fresh or dried according to the forms of use desired through infusion, decoction, powder or smoking. Nuts are processed into oil and soap. Soap (45%) and oil (30%) were the most common forms of use adopted by the local communities (Figure 7). There were variations in CMU among ethnic and gender groups (Tables 7 and 8). The CMU showed that oil uses were higher for women (0.33) than for men (0.28). Women showed a higher consensus value for soap uses (0.50 and 0.57) than men (0.34 and 0.44) respectively, in Ziékorodougou and Niankorobougou. The lowest CMU were observed for soap use by the Senufo (0.39) and the men (0.34) in Ziékorodougou. Leaves, bark and roots of *Carapa procera* were exploited and used as an infusion and decoction. In both locations, the use of decoctions has been more significant for men (CMU = 0.13 and 0.16) than for women (CMU = 0.04 and 0.09). Smoking of dried gum for magico-spiritual uses has been reported by the Senufo ethnic group.

**Table 7:** **Consensus values (CMU) for forms of use from** ***Carapa procera*** **according to the ethnic groups**

Form of use	Ziékorodougou		Niankorobougou	
	Senufo	Fulani	Bambara	Fulani
Infusion	-	-	0.05	-
Decoction	0.11	0.07	-	0.22
Smoking	0.04	-	-	-
Bath/shower	0.04	0.11	-	-
Oil	0.28	0.32	0.32	0.29
Raw dry	0.04	-	-	-
Powder	0.04	-	-	-
Soap	0.39	0.43	0.63	0.41
Firewood	0.07	0.07	-	0.07

**Table 8:** **Consensus values (CMU) for forms of use from** ***Carapa procera*** **by gender**

Form of use	Ziékorodougou		Niankorobougou	
	Men	Women	Men	Women
Infusion	-	-	0.04	-
Decoction	0.13	0.04	0.16	0.09
Smoking	0.03	-	-	-
Bath/shower	0.09	0.04	-	-
Oil	0.28	0.33	0.28	0.33
Raw dry	0.03	-	-	-
Powder	0.03	-	-	-
Soap	0.34	0.50	0.44	0.57
Firewood	0.06	0.08	0.08	-

**Figure 8:**
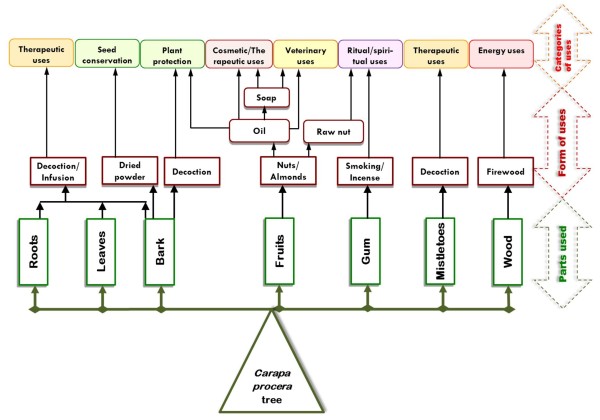
**Sociocultural values for** ***Carapa procera*** **.**

Figure 8 show the sociocultural values for *Carapa procera* plant parts.

### Reasons of use

The treatment of skin diseases, gastrointestinal, body care, animal healthcare and phytosanitary treatment of plants are among other, the reasons to use *Carapa procera*. Tables 9 and 10 show the FL of use of *Carapa procera* according to the ethnic groups and the gender of informants. The uses for body care and household sanitation have received the greatest level of fidelity. Wound and injury care treatment of gastrointestinal diseases and treatment of skin diseases have also been common reasons of use. The Senufo ethnic group indicated the use of dried powder for agricultural seed conservation and burying of whole seeds to field boundaries for crop protection. Insecticide treatment of crops was mentioned by the Senufo and Fulani ethnic groups in Ziékorodougou.

**Table 9:** **Fidelity level (FL) of reasons of use of** ***Carapa procera*** **according to the ethnic groups**

Reasons of use	FL (%)			
	Ziékorodougou		Niankorobougou	
	Senufo	Fulani	Bambara	Fulani
Treatment of skin deseases	33	42	8	17
Treatment of malaria	-	8	8	17
Treatment of onchocerciasis	17	-	-	33
Treatment of hemorrhoids	-	-	-	17
Treatment of gastrointestinal deseases	33	33	-	42
Care of lesions. wounds and injuries	17	42	42	50
Protection against mosquitoes and parasites	8	-	-	8
Body care and household sanitary	83	92	100	92
Invigorating relaxing and body massage	17	8	17	33
Animal care	-	8	8	17
Insecticide treatment of crops	8	17	-	-
Seed conservation	8	-	-	-
Household fuel	17	17	-	17
Protection of crop fields	8	-	-	-
Wishes and prayers (magico-mystics)	8	-	-	-

**Table 10:** **Fidelity level (FL) of reasons of use of** ***Carapa procera*** **by gender of informants**

Reasons of use	FL (%)			
	Ziékorodougou		Niankorobougou	
	Men	Women	Men	Women
Treatment of skin deseases	58	17	8	17
Treatment of malaria	8	-	25	-
Treatment of onchocerciasis	8	8	25	8
Treatment of hemorrhoids	-	-	8	8
Treatment of gastrointestinal deseases	33	33	25	17
Care of lesions. wounds and injuries	33	25	42	50
Protection against mosquitoes and parasites	8	-	8	-
Body care and household sanitary	83	92	92	100
Invigorating relaxing and body massage	-	25	33	17
Animal care	8	-	8	17
Insecticide treatment of crops	25	-	-	-
Seed conservation	8	-	-	-
Household fuel	17	17	17	-
Protection of crop fields	8	-	-	-
Wishes and prayers (magico-mystics)	8	-	-	-

Informal interviews revealed that the powder of dried nuts in combination with other products has aphrodisiac virtues. The hulls of fruits are used as anti-inflammatory for the treatment of swelling.

## Discussion

This study showed that *Carapa procera* is a species with multiple uses and high use values and that different plant parts are used by local people for various reasons. All plant parts (roots, wood, bark, nuts, leaves and mistletoes) are exploited. A high diversity in the use of plant parts from *Carapa procera* was also reported by Guèye et al. [34]. Nuts are the product of main importance, as also confirmed by Weber et al. [32]. Similar results were reported by Koura et al. [38], who found that the seeds of the fruit of *Parkia biglobosa* were the most used product. In our study, nuts constituted 57% of the citations of exploited plant parts. Women have showed a higher consensus value for nuts than men. This reflects the importance of nuts in women’s activities. The CPP for bark harvesting was similar for both sexes in Niankorobougou, but it was higher for men than for women in Ziékorodougou. These results are comparable to those in Schumann et al. [39] that showed that the baobab fruit was more important for women, while the bark was more important for men. Ziékorodougou men showed the highest level of knowledge of plant parts exploited.

The UD and UE showed a high proportion of cosmetic and therapeutic uses. These results confirm those in Guèye et al. [34], who reported that the main uses of *Carapa procera* are medicinal. This importance of the medicinal uses of local plants has also been reported in previous studies [28, 33]. According to ethnic groups, Bambara showed the highest consensus value for cosmetic uses. This significance accorded to cosmetic uses was confirmed by the higher consensus value for soap use. Comparatively, the Fulani showed significant importance for veterinary uses. This observation is consistent with their traditional role in the management of animals. Related to gender, the use diversity values showed that cosmetic uses were higher for women than for men. This has resulted in the highest consensus value for oil and soap uses by women.

Soap and oil were the most common forms of use adopted by the local communities. A lack of opportunities for selling oil at the markets leads producers to transform oil into soap for household uses. This form of use justifies the high proportion of specific reasons of uses related to body care, household sanitary and hygiene. In both locations, soap has been more important for women than for men. This observation is in accordance with the traditional role of women in household health and hygiene (washing dishwashing and laundry). The use of decoctions has been more significant for men than for women. This is consistent with the most common use of the bark and roots by men to their specific needs.

Plant parts of *Carapa procera* are used variously in traditional human medicine, cosmetics (soap and skin care), veterinary medicine, insecticide treatment of plants, for magico-spiritual purposes and energy. Lykke et al. [12] showed that the importance attached to a species depends on the different categories of use by the people. However, Benz et al. [40] showed that these uses can sometimes change rapidly, depending on market opportunities. This could be the case of *Carapa procera* oil, which was sold by the producer at 1500–2000 FCFA (2.29 – 3.05 Euros) per liter in the study area. In the context of market development, the sale of oil would be a significant source of income for households, particularly for women.

The reasons of uses showed that body care and household sanitation have scored the highest FL value followed by wound and injury care, treatment of skin diseases and gastrointestinal diseases. Treatment of onchocerciasis and protection against mosquitoes and other pests were also cited. These specific reasons of uses accord with the work of Sylla et al. [41] that have shown that traditional repellents based on “*kobi*” can be an effective aid against the blackfly nuisance. Insecticide treatment of crops was mentioned by the Senufo and Fulani ethnic groups in Ziékorodougou. The use of *Carapa procera* oil for phytosanitary treatment of organic crop as cotton has also been reported by Weber et al. [32].

In a biological perspective, pharmacological and phytochemical studies can be carried out for extensive research on plant parts and medicinal properties of *Carapa procera* products.

In this study, local knowledge on *Carapa procera* species were diversified and varied according to ethnic group. The differences in use values between ethnic and gender groups can be explained by the social division of labor in the community. This is consistent with the works of Gouwakinnou et al. [42] that showed that the difference in use values is not necessarily related to ethnicity. This is illustrated in our study by the fact that the Fulani who were represented in the two study locations did not show similarity in the use values.

## Conclusion

The present study has provided information about use-value and socio-cultural knowledge on *Carapa procera* trees. The assessment of use diversity value (UD) has highlighted the importance of *Carapa procera* through its use categories and confirms its status as multipurpose species. Local knowledge on *Carapa procera* were diversified and varied according to ethnic group and gender. Among the ethnic groups studied, the Senufo ethnic group showed the highest level of knowledge about used parts and forms of use on *Carapa procera*. Cosmetic and therapeutic uses have proven to be the most important in the study zone showing a potential for *Carapa procera* oil for industrial uses. Veterinary uses of the species also offer opportunities for pharmacological research to help communities to improve animal healthcare services.

Despite the multipurpose uses of *Carapa procera*, its products are undervalued. Commercial use of oil is low in relation to its potential value in farming, cosmetic and pharmaceutical industries. In perspective, the promotion and enhancement of *Carapa procera* can provide significant socio-economic benefits to local people. In this context, it is necessary to implement strategies to support local communities to actively participate in the conservation and sustainable use of the species as part of the preservation of plant biodiversity.
